# Diagnostic accuracy of sensory and motor tests for the diagnosis of carpal tunnel syndrome: a systematic review

**DOI:** 10.1186/s12891-021-04202-y

**Published:** 2021-04-07

**Authors:** Armaghan Dabbagh, Joy C. MacDermid, Joshua Yong, Tara L. Packham, Luciana G. Macedo, Maryam Ghodrati

**Affiliations:** 1 School of Physical Therapy, Faculty of Health Science, Elborn College, Western University, London, ON Canada; 2 School of Rehabilitation Science, McMaster University, Hamilton, ON Canada; 3 Roth McFarlane Hand and Upper Limb Centre, St. Joseph’s Hospital, London, ON Canada; 4 Sengkang General Hospital, Singapore, Singapore

**Keywords:** Carpal tunnel syndrome, Diagnostic accuracy, Sensory and motor tests, Systematic review

## Abstract

**Background:**

Carpal tunnel syndrome (CTS) is the most common entrapment mononeuropathy of the upper extremity. The previous systematic review of the diagnostic tests for CTS was outdated. The objective of this study was to compile and appraise the evidence on the accuracy of sensory and motor tests used for the diagnosis of CTS.

**Methods:**

MEDLINE, CINAHL, and Embase databases were searched on January 20, 2020. Studies assessing at least one diagnostic accuracy property of the sensory or motor tests for CTS diagnosis were selected by two independent reviewers. Diagnostic test accuracy extension of the PRISMA guidelines was followed. Risk of bias and applicability concerns were rated using QUADAS-2 tool. Any reported diagnostic accuracy property was summarized. Study characteristics and any information on the accuracy of the sensory and motor tests for CTS diagnosis were extracted.

**Results:**

We included sixteen clinical studies, assessing thirteen different sensory or motor tests. The most sensitive test for CTS diagnosis was the Semmes-Weinstein monofilament test (with 3.22 in any radial digit as the normal threshold) with sensitivity from 0.49 to 0.96. The tests with the highest specificity (Sp) were palmar grip strength (Sp = 0.94), pinch grip strength (Sp from 0.78 to 0.95), thenar atrophy (Sp from 0.96 to 1.00), and two-point discrimination (Sp from 0.81 to 0.98).

**Conclusions:**

The evidence was inconclusive on which sensory or motor test for CTS diagnosis had the highest diagnostic accuracy. The results suggest that clinicians should not use a single sensory or motor test when deciding on CTS diagnosis.

**Trial registration:**

PROSPERO CRD42018109031, on 20 December 2018.

## Background

Carpal Tunnel Syndrome (CTS) is the most common compression neuropathy of the upper extremity, happening as the results of median nerve entrapment in the carpal canal [1]. Persons with CTS have sensory or motor problems in the area innervated by the median nerve [2]. The prevalence of CTS has been estimated to be 4–5% in the general population, with a higher prevalence in the working population [3].

In its latest guideline, the American Association of Orthopedic Surgeons (AAOS), has categorized CTS clinical diagnostic tests in four main categories: 1) provocative maneuvers (e.g. Durkan’s test, Phalen’s test), 2) sensory and motor tests (e.g. heat/cold sensation, thenar muscles atrophy), 3) questionnaires and scales (Boston carpal tunnel questionnaire, CTS-6 scale), and 4) hand symptoms diagrams/maps (such as Katz and Stirrat’s hand symptoms diagram) [4]. Advantages of clinical diagnostic tests include that they can be done quickly, do not cost much, are not painful, and yield immediate results.

A systematic review (SR) of the diagnostic accuracy of clinical examination tests was conducted by one of our research team members in 2004 and is currently outdated [5]. Several original studies have been published after 2004 that were not included in any other reviews in the past 16 years [6–11]. This paper is one of a series of updated SRs related to the diagnostic accuracy of CTS clinical diagnostic tests categorized by the AAOS. We previously published an SR of scales, questionnaires, and hand symptom diagrams [12]. The focus of this SR is on sensory and motor tests, and we aimed to identify, critically appraise and synthesize the evidence on the diagnostic accuracy of the sensory and motor tests for diagnosing CTS in individuals with suspected CTS.

## Methods

We registered the protocol of this SR on December 20, 2018 with the International Prospective Register of Systematic Reviews (PROSPERO), with the registration number of CRD42018109031 [13]. We followed the Diagnostic Test Accuracy extension of the Preferred Reporting Items for Systematic Reviews and Meta-Analyses (DTA-PRISMA) [14] and the Cochrane collaboration guidelines in developing and reporting this SR [15].

### Information sources

We conducted a systematic computerized search of Medline and Embase through Ovid, as well as CINAHL, all from inception until January 20, 2020. We developed our search strategy in consultation with a health science research methodologist librarian at McMaster University in two meetings. We originally developed a search strategy that captured all the four components of the clinical diagnostic tests outlined by the AAOS. However, due to the large number of study results and the variety of identified tests, we only focused on sensory and motor tests in this SR to increase the ease of readability for the target audience. Our search strategy included search terms for three main concepts including CTS, diagnostic accuracy properties, and names of the diagnostic tests for CTS. The search strategy can be found in Appendix A.

### Study selection

Two authors (AD, JY) independently selected studies in two consecutive phases. In the first phase of study selection, titles and abstracts of the included citations were reviewed based on a pre-determined set of eligibility criteria. In order to enhance the quality of the review process, AD and JY initially reviewed 100 of the citations and resolved their disagreements through discussions. In the second phase, after retrieving the full texts of the included articles, two authors again independently assessed the eligibility of the articles for inclusion in this SR. The kappa agreement between the authors in the first phase of screening (titles and abstracts) was calculated using STATA statistical analysis software, version15 [16]. Kappa values below 0.20 suggest poor agreement, and values of larger than 0.80 indicate perfect agreement [17]. Any disagreements between AD and JY in the process of study selection was resolved by the most experienced research team member (JM) through discussion.

### Eligibility criteria

We did not exclude any studies based on their language, sample size, choice of reference standard, or gender of the included participants. We included studies that met the following inclusion criteria.

#### Design

We included case-control, cross-sectional, and cohort (both retro- and prospective) study designs that were in a full-report format.

#### Participants

We included studies on persons who were diagnosed or suspected to have CTS and were older than 18 years old. The studies must have had a control group of people diagnosed with any type of upper limb musculoskeletal, neurological, or vascular conditions, such as cervical radiculopathy, or De Quervain’s tenosynovitis. We excluded studies that had healthy control groups, as healthy control groups would falsely inflate the diagnostic accuracy properties and are not reflective of the actual clinical settings.

#### Diagnostic test

Studies that assessed the diagnostic accuracy of at least one sensory or motor test for CTS diagnosis.

#### Comparison

Since there is no gold standard for CTS diagnosis, we decided to accept studies with any reference standard, ranging from electrodiagnosis testing, to carpal tunnel release surgery and clinical examination tests.

#### Outcome

We included articles that reported at least one diagnostic accuracy property, such as sensitivity (Sn), Specificity (Sp), positive predictive value (PPV), negative predictive value (NPV), or articles providing enough data on their test results enabling us to (re)synthesize 2 × 2 contingency tables.

#### Time

Any time frame reporting diagnostic accuracy of the sensory or motor tests for CTS diagnosis.

### Data extraction

Initially MG and AD extracted data from three of the included studies, and since the agreement was high, MG did the remainder of the extraction independently, and AD cross checked the information. We used a self-developed, pre-determined extraction sheet previously developed to extract information for a SR of diagnostic accuracy of scales, questionnaires and hand symptom diagrams for CTS diagnosis. We extracted the following data:

1. Information about the studies, such as authors, study design, year and country, conflicts of interest.
2. Information on the participants, such as sample size, age, gender, inclusion and exclusion criteria, diagnoses, severity and duration of symptoms, and CTS prevalence in the sample.
3. Information regarding the index test, index test methodology and threshold criteria for positive results, as well information on the reference standard.
4. Any information on the diagnostic accuracy properties of the sensory or motor tests, such as Sn, Sp, NPV, PPV.

### Data synthesis and analysis

We extracted information on Sn, Sp, PPV, NPV, positive likelihood ratio (+LR), negative likelihood ratio (−LR) and their associated 95% confidence intervals (95%CIs) from the included studies, where possible. When this information was not directly reported in the studies, we tried to calculate them by reconstructing 2 × 2 contingency tables based on the available data on true and false positives and negatives.

PPV and PPV are affected by the prevalence of the condition in the sample, for instance, an increase in the prevalence of a given condition in a sample increases the PPV and decreases the NPV [18]. To overcome the previously mentioned issues associated with NPV and PPV, we tried to calculate and report +LR and -LR, where possible. Likelihood ratios are independent from the prevalence of the condition in the sample and provide a more accurate clinical judgment [18]. Following is an interpretation of the likelihood ratios: +LR > 10, and -LR < 0.1 indicate a great change in the posttest probability and are very valuable in the clinical decision-making process [18]. + LR of 5 to 10 and -LR of 0.1 to 0.2 indicate a moderate change in the posttest probability of having a condition [18]. + LR of 2 to 5, and -LR of 0.2 to 0.5 indicate slight change in the posttest probability [18]. Lastly, +LR <  2 and -LR > 0.5 have no clinical value in calculating the posttest probability [18].

We categorized and presented the information on the diagnostic accuracy of the sensory and motor tests for CTS diagnosis in separate tables. The results were grouped into ‘sensory tests for CTS diagnosis’ and ‘motor tests for CTS diagnosis’, with each category organized by the frequency of the diagnostic test being assessed. Due to the heterogeneity of the data (different sample characteristics, different index and reference tests methodology and criteria for positive results) we could not conduct a meta-analysis.

### Assessment of risk of Bias and applicability concerns

Two authors (AD, JY), independently rated the risk of bias and applicability concerns of the included studies based on the revised tool for the quality assessment of diagnostic accuracy studies (QUADAS-2) [19]. In case of any disagreements in rating the quality of the studies, a third research team member (JM) was engaged and the disagreement was resolved through discussion. The QUADAS-2 tool assesses risk of bias in four domains: a) patient selection, b) index test, c) reference standards, and d) study flow and timing [19]. Moreover, QUADAS-2 rates the applicability concerns in three domains addressing patient selection, index test, and reference standard [19].

## Results

**Fig. 1:**
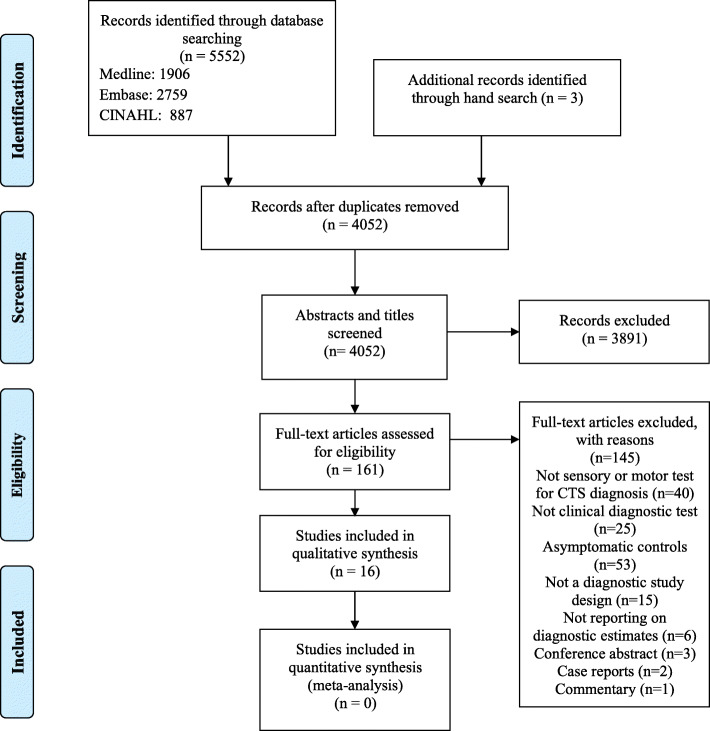
PRISMA diagram

We identified 5552 citations through the electronic database search. After removing the duplicates, we reviewed the titles and abstracts of 4052 citations. In the second phase of screening, we reviewed the full texts of 161 articles, of which 16 articles were included in this SR (Fig. 1. PRISMA diagram). The reviewers had a kappa agreement of 0.70 (SE: 0.02, 95% CI = [0.66–0.74]) in screening the titles and abstracts. The studies were conducted in USA, Sweden, France, Canada, Spain, Portland, Italy, and Turkey. Appendix B summarises the reported conflict of interests of the included studies. The characteristics of the included studies are presented in Table 1. All of the studies had prospective cross-sectional designs, except for two studies that had retrospective designs [6, 23], and one that had prospective cohort design [11].

**Table 1:** Study characteristics

Author/year/ study design	Country	n-hands	n-individuals	n-CTS	Diagnostic tool(s)	Reference standard
Borg & Lindblom [20] /1988/ Prospective cross-sectional	Sweden	33	22	22	QST consisted of vibrometry, tactile thresholds, Von Frey hairs, 2PD, graphesthesia, warm and cold thresholds	A combined battery of tests
Buch-Jaeger & Foucher [21] / 1994/ Prospective cross-sectional	France	172	112	112	Vibratory sensibility, static 2PD, SWMFs	NCS
Dale et al. [8] /2011 / Prospective cross-sectional	USA	NR	1108	NR	SWMFs	Modified Katz Hand Diagram; NCS; The Consensus Criteria CTS Case Definition
Franzblau et al. [22] /1993/ Prospective cross-sectional	USA	260	130	22	Vibration, hand grip strength, palmar pinch grip	NCS; NCS+ surveillance symptom definitions for CTS; Physical examination + surveillance symptom definitions for CTS
Katz et al. [23] /1990/ Retrospective cross sectional	USA	165	110	44	2PD	NCS
Kucukakkas & Yurdakul [7]/ 2019/ Prospective cross-sectional	Turkey	463	463	226	Thenar atrophy, 2PD	NCS
Kuhlman et al. [24] /1997/ Prospective cross-sectional	USA	228	143	142	Hypoesthesia, APB weakness	NCS
MacDermid et al. [25] /1994/ Prospective cross-sectional	Canada	78	39	23	SWMFs	Clinical profile of CTS + NCS
MacDermid et al. [26] /1997/ Prospective cross-sectional	Canada	84	42	36	Vibration, pinch grip strength, SWMFs	NCS + positive clinical examination
Makanji et al. [11] /2013/ Prospective cohort	USA	NR	88	65	Thenar atrophy, thumb abduction weakness	NCS
Naranjo et al. [9] /2007/ Prospective cross-sectional	Spain	105	68	80	Thenar atrophy	NCS
Pagel et al. [27] /2002/ Prospective cross-sectional	Portland	NR	113	60	SWMFs	NCS
Raudino [28] /2000/ Prospective cross-sectional	Italy	140 hands	83	83	Hypoesthesia, thumb abduction Weakness	NCS
Sartorio [10] /2017/ Prospective cross-sectional	Italy	NR	80	80	Functional dexterity test	NCS
Szabo et al. [29] /1999/ Prospective cross-sectional	USA	CTS = 87 other diagnoses of UE = 90	100	87	SWMFs, grip strength	NCS
Yildirim & Gunduz [6] /2015/ Retrospective cross-sectional	Turkey	124	62	49	SWMFs	NCS

List of abbreviations: *ROB* risk of bias, *2PD* two-point discrimination, *SWMFs* Semmes-Weinstein monofilaments, *APB* Abductor Pollicis Brevis, *NCS* nerve conduction studies, *QST* quantitative sensory testing, *NR* not reported, *CTS* carpal tunnel syndrome, *ROB* risk of bias

Thirteen sensory or motor tests were assessed in the included studies, which were Semmes-Weinstein monofilament (SWMFs)(*n* = 7), vibrometry (*n* = 4), hand grip strength (*n* = 2), pinch grip strength (*n* = 2), thumb abduction weakness (*n* = 3), functional dexterity test (*n* = 1), thenar muscle atrophy (*n* = 3), hypoesthesia (*n* = 2), two-point discrimination (*n* = 4), tactile thresholds (*n* = 1), Von Frey hairs (*n* = 1), warm and cold thresholds (*n* = 1), and graphesthesia (*n* = 1). A description of these tests, as well as their method of conduction and positive tests criteria are presented in Table 2.

**Table 2:** Description of Sensory/Motor Tests for Carpal Tunnel Syndrome diagnosis (sorted alphabetically)

Diagnostic Test	Method	Positive Result Threshold
**Functional dexterity test** [10]	• Was administered independently on both sides. The task was to overturn all the pegs using only the movement of the first three fingers of a hand (without supinating or pronating the forearm or resting the elbow) starting from the top and the opposite side from the hand with which the test is performed. At the next time taken to complete the test, 5 s of penalties were added each time the patient pronated the forearm or touched the edge of the hole with the peg, and 10 s of penalty if the patient dropped the peg [10].	• If the total time is greater than the value corresponding to the 97th percentile of the normative data of the healthy Italian population, corrected by sex and age class [10].
**Graphesthesia** [20]	• A figure was written on the finger pad with a blunt pencil [20]	• The threshold was defined as the height in mm of the smallest figure that was identified by the patient [20].
**Hand grip strength** [22, 29]	• Measured using a Jamar Hydraulic Hand Dynamometer (J.A. Preston Corporation, Jackson, Michigan) [22] • Measured using either the Jamar dynamometer (Preston, Jackson, MI) or the Greenleaf Solo System (Palo Alto, CA). Grip was measured at each setting (I to V). Key (side-to-side) pinch, 3-jaw (tripod) pinch, and tip-to-tip pinch strengths were also measured using the Greenleaf Solo System. Each test was performed 3 times and the resultant mean values were used for data analysis [29].	• Hand grip and palmar pinch grip results were considered abnormal if they were more than 1.65 standard deviations below the mean for persons of the same age and sex [22]. • Evaluated grip strength by comparing subjects’ right hands with their left hands. They considered strength diminished if grip strength at position III on the dynamometer was more than 12% less on the affected side than the contralateral side. The same assumptions were applied to key pinch, 3-jaw (tripod) pinch, and tip-to-tip pinch strengths [29].
**Hypoesthesia** [20, 24, 28]	• The sensibility screening was carried out with cotton wool, pins and warm and cold metallic rollers (40 °C and 20 °C, respectively) [20]. • A pinwheel was rolled across the palmar aspect of the index and small fingers [24]. • The sensitivity was evaluated by perception of pinprick [28]	• The test was considered positive if the subject reported hypesthesia of the index finger compared with the small finger [24].
**Pinch grip strength** [22, 26]	• Measured with a B&L Pinch Gauge (B&L Engineering, Santa Fe Springs, California) [22] • The pinch was performed by having the patient actively pinch a piece of paper between the tips of the thumb, index and long fingers using MP flexion and IP extension [26].	• Hand grip and palmar pinch grip results were considered abnormal if they were more than 1.65 standard deviations below the mean for persons of the same age and sex [22]. • If symptoms reproduced within 60 s [26].
**Semmes-Weinstein monofilament testing (SWMFs)** [6, 8, 21, 25–27, 29]	• The 20-piece kit of SWMFs (North Coast Medical, San Jose, CA) was used to test sensory thresholds of the tips of the thumb, the index finger, and the long and small fingers using standard clinical techniques. Monofilaments were applied three times, with a positive response in one or more of the applications indicating that the stimulus was perceived [25]. • SMWs was done on the distal palmer pad of each digit of the hand in with enough force to bow the monofilament for a total of 1.5 s. The monofilaments were applied three times, with a positive response to one or more of the applications indicating that the stimulus was perceived [27]. • The monofilament was applied 3 times to each digit and the palm; a patient’s affirmative response to 1 or more of the monofilament applications indicated the stimulus was perceived. The monofilament kit contains 5 monofilaments to mark 5 selected thresholds: 2.83 (normal), 3.61 (diminished light touch), 4.31 (diminished protective sensation), 4.56 (loss of protective sensation), and 6.65 (loss of deep pressure sensation). The numeric value represents the logarithm of 10 times the force in milligrams required to bow the monofilament. All subjects were tested with their wrists in neutral position. The tests were then repeated after the subjects held their wrists flexed (Phalen’s maneuver) for 5 min [29].	• Recorded thresholds were categorized as normal or abnormal using four decision rules and two criterion measures. The decision rules were (1) a threshold higher than 2.83, (2) a threshold higher than 2.83 and higher than the threshold of the small finger (D5), (3) a threshold higher than 3.22, and (4) a threshold higher than 3.22 and higher than the threshold of the small finger. The two criterion measures were (1) the highest threshold of the three radial digits (D1-D3) and (2) the threshold of the long finger alone (D3) [25]. • A classification of abnormal was assigned if the SWMF threshold for any of the radial three digits was greater than 2.83 and greater than the threshold for the small finger [6, 26]. • Two separate sets of criteria: • SWM 1: a positive test was defined as stimulus perception by the patient in any one of the radial three digits at a threshold value of 2.83 or an absent stimulus perception. • SWM 2: a positive test was defined by stimulus perception at threshold value of 2.83 or an absence of stimulus perception using only digit 3 and using digit 5 for internal comparison. • The patient must have had a digit 3 SWM test of 2.83 and a digit 5 test of 2.83 [27].
**Tactile thresholds** [20]	• Pulses consisted of half sinusoids of 100 Hz from a Bruel & Kjaer shaker and were applied perpendicularly to the skin of the finger pads via a 2 mm diameter blunt plastic probe. The amplitude of the stimulus pulse was increased or decreased in small increments [20].	• The lowest amplitude that was felt in at least three of four consecutive stimulations was taken as the “yes response”, and the lowest amplitude that was not felt in 3 of 4 stimulations as the “no response”. The threshold was defined as the average of these 2 values [20].
**Thenar atrophy**^9,307^	• Thenar atrophy was defined as concavity of the thenar muscle group along the plane parallel to the palm and was scored as either present or absent [7].	• No description
**Thumb abduction weakness** [24, 28, 30]	• The subject placed the touch pads of the thumb and small finger together. The examiner then applied a strong posteriorly directed force at the thumb interphalangeal joint toward the metacarpophalangeal joint of the index finger while instructing the subject to give maximum effort to keep the touch pads together [24]. • The strength of the abductor pollicis ensuring that the thumb was parallel to the index finger and the movement was occurring at the metacarpal trapezial joint [28].	• The test was positive if any weakness was detected [24].
**Two-point discrimination** (2PD)^20,21,267^	• The gap was successively decreased between the 2 points of a pair of blunted dividers, applied perpendicularly to the pulp of the finger [20]. • Static 2PD Tested on the pulp of the index finger using the Disk-criminator [21]. • Moving (dynamic) 2PD with electrocardiogram calipers with tips set 4 mm apart. The index and fifth fingertips were stroked five times with either one or two caliper tips [23]. • Two-point discrimination was performed in order to determine sensory loss. The Dellon discriminator was used on the index and third-finger fingertips [7].	• The threshold was defined as the smallest gap in mm at which the patient could identify that there were 2 points [20]. • The normal being taken as less than 6 mm [21]. • Failure to identify correctly the number of points on two or more strokes was considered abnormal [23]. • Greater or equal to 6 mm was accepted as altered sensation [7].
**Vibrometry** [20–22, 26]	• A 100 Hz sine wave was produced by an electromagnetic vibrator. The peak to peak vertical movement of the 13 mm diameter blunt stimulus probe was recorded continuously in microns by means of an accelerometer. The variable tissue damping of the vibration amplitude was thus excluded as a source of error [20]. • Tested by the application of a branch of a tuning fork (256 cycles per second) to the pulp of the index finger and comparing the perceived intensity to that in the little finger in the same hand [21]. • Determined in the 2nd finger of each hand with a Vibratron II (Physitemp, Clifton, New Jersey) using a standard psychophysical technique and published normal values based on age and height [22]. • Testing with the prong of a 256 cycle per second tuning fork was performed on the fingertip [26]	• The perception threshold was determined with the method of limits, i.e. as the average of appearance and disappearance thresholds when the stimulus was successively increased and decreased. Vibratory threshold was determined at least 3 times at each site and the mean was calculated [20]. • A vibratory threshold was considered abnormal if it was more than 1.65 standard deviations above the mean for persons of that age and height [22].
**Von Frey hairs** [20]	• A series of 10 nylon filaments of different diameters and length with log arrhythmically spaced bend pressures from 0.02 to 10 g were applied perpendicularly to the pulp of the finger. Each filament was applied 10 times at irregular intervals (to avoid the error of rhythmical response).	• The threshold was defined as the pressure which was felt closest to half of the 10 stimulations [20].
**Warm and cold thresholds** [20]	• Determined according to Fruhstorfer et al. (1976) [20].	• No description

Participants’ characteristics are summarized in Table 3, including their age, gender, duration and severity of symptoms, sampling method, process of selection, and eligibility criteria. Overall, 2763 individuals were included in these studies, of whom 1131 had CTS.

**Table 3:** Participants’ characteristics table

Study	Age/ gender/ sample	Severity of CTS/ Duration of symptoms	Process of participants selection	Inclusion and exclusion criteria
**Borg & Lindblom 1988** [20]	Mean = 48, Range = 20–71/ 86.4% W, 13.6% M/ consecutive	NR/ Mean = 1 year, Range = 2 months to 22 years	Patients referred to either the department of Neurology or the department of Clinical Neurophysiology,	IC: Sensory or motor symptoms from the median nerve territory distal to the wrist; 2) Positive “wrist flexion test” as described by Phalen and/or significant nerve conduction or electromyographic abnormalities consistent with compression of the median nerve at the wrist level. EC: NR
**Buch Jaeger & Foucher 1994** [21]	Mean = 52, Range: 29–81/ 80% W, 20% M/ consecutive	NR/ Mean = 26 months, Range 1–120 months	112 patients presenting with signs of carpal tunnel syndrome, 60 of them bilaterally, were referred for nerve conduction studies.	IC: Paresthesia in the territory of the median nerve in the hand 2) Occasional pain 3) Nocturnal recrudescence of the symptoms 4) Numbness leading to clumsiness of the hand. EC: NR
**Dale et al. 2011** [8]	Mean = 30.3; SD = 10.3/ 35% W 65% M/ consecutive	NR/ NR	Subjects were recruited from eight employers and three construction trade union apprenticeship programs between July 2004 and October 2006.	IC: > 18 years and starting a new full-time job (over 30 h per week) or changing their work benefits status. EC: 1) If they had a current or previous diagnosis of CTS or peripheral neuropathy 2) contraindication to NCS or pregnant.
**Franzblau et al. 1993** [22]	Mean = 34.1, SD = 11/ 56.% W 44% M/ consecutive	NR/ NR	All workers in the plant were invited to participate in the medical survey.	NR
**Katz et al. 1990** [23]	Mean = 45.6, SD = 14.4/ 66.4% W 33.6% M/ consecutive	NR/ < 2 months = 21 subjects 2 to 12 months = 42 subjects > 12 months = 44 subjects	Patients with upper extremity complaints of diverse causes referred to a neurophysiology laboratory for diagnostic studies. Eligible patients were identified by review of the laboratory schedule	IC: Patients referred to the Brigham and Women’s Hospital Neurophysiology Laboratory for electrophysiologic evaluation of upper extremity complaints EC: Under 18 years old
**Kucukakkas & Yurdakul 2019** [7]	Mean = 46.7, SD = 12.7/ 20.7% M79.3% W/ consecutive	Negative = 51.1% Minimal = 0.9% Mild = 12.1% Moderate = 24.8% Severe = 10.2% Extreme = 0.9% / 9 ± 6.4 months	Patients who visited an outpatient clinic, with symptoms consistent with CTS	IC: Paresthesia or pain in the median nerve distribution of the hand, existed for at least 3 months EC: Pregnancy, prior history of wrist fracture or surgery, cervical radiculopathy, polyneuropathy or mononeuropathies
**Kuhlman et al. 1997** [24]	NR /NR / consecutive	NR/ NR	Subjects referred for electrodiagnostic consultation with suspected CTS were evaluated.	IC: Subjects had at least one symptom indicative of possible CTS EC: 1) generalized peripheral neuropathy, 2) previous carpal tunnel surgery, 3) cervical radiculopathy, or 4) some other neuromuscular disorder that could account for their signs and symptoms. Subjects with diabetes were not excluded unless their NCSs demonstrated a generalized peripheral neuropathy.
**MacDermid et al. 1994** [25]	CTS patients: Mean = 47, SD = 15; Non-CTS patients: Mean = 31, SD = 13 / NR/ consecutive	Mild = 36% Moderate = 36% Severe = 28%/ NR	New patients who had been referred to the hand clinic with complaints of numbness, tingling, and/or pain affecting one or both hands.	IC: New referred patients with complaints of numbness, tingling, and/or pain affecting one or both hands. EC: 1) Ulnar neuropathies 2) Their small fingers were not considered legitimate comparators 3) Overuse-related diagnoses, such as tendinitis, and nonspecific repetitive strain injury
**MacDermid et al. 1997** [26]	Mean = 47, SD = 15/ NR/ consecutive	Mild = 36% Moderate = 35% Severe = 28% / NR	Patients referred to the clinic with a history of gradual onset of pain, numbness or tingling	IC: Patients referred to the clinic with a history of gradual onset of pain, numbness or tingling EC: 1) Acute injuries, 2) previous upper extremity surgery, 3) complaints related to congenital malformations, 4) dupuytren’s disease, 5) tumors, 6) severe hand deformities.
**Makanji et al. 2013** [11]	Mean = 56, Range = 21–85 / 62% W 38% M/ consecutive	Mild = 7% Moderate = 44% Severe = 23% / NR	Adult patients in the practice of four hand surgeons that were prescribed electrophysiological testing to diagnose suspected CTS were invited to enrol.	IC: Adult patients in the practice of four hand surgeons that were prescribed electrophysiological testing to diagnose suspected CTS were invited to enrol EC: 1) Prior carpal tunnel release, 2) Prior diagnosis of CTS, 3) Median nerve surgery, 4) Previous electrophysiological testing of the median nerve, 5) Rheumatoid arthritis, and 6) Pregnancy
**Naranjo et al. 2007** [9]	Mean = 47, SD = 11/ 56 W 12 M/ consecutive	Mild = 13 hands Moderate = 30 hands Severe = 37 hands/ Mean duration = 21 months, Interquartile Range = 8–36	Adult patients with suspected CTS referred to the outpatient Rheumatology clinic at the University Hospital Dr. Negrin in Las Palmas, Spain, between December 2005 and May 2006 were selected for the study.	IC: Sensory symptoms over the distribution of the median nerve regardless of the results of Phalen’s or Tinel’s maneuvers. Also, burning pain or numbness aggravated by sustained positions and relief by shaking or moving the hands, sleep disruption by symptoms, and daily complaints over at least a three-month period EC: 1) Had undergone surgery, or 2) traumatic injuries at the target wrist, 3) hypothyroidism, acromegaly, 4) polyneuropathy or radiculopathy, 5) pregnancy, 6) fibromyalgia, 7) rheumatoid arthritis or crystal arthritis or 8) had received injections, or 9) presented ganglions, tenosynovitis or arthritis
**Pagel et al. 2002** [27]	Mean = 52.8, Range = 23–85, SD = 13.7/ 7.1% W 92.9% M/ consecutive	NR/ NR	Patients were referred to the electrodiagnostic laboratory of the Portland Veterans Affairs Medical Center between August 5, 1999, and June 1, 2000, for evaluation of symptoms suggestive of CTS.	IC: Symptoms of paresthesia inclusive of the median nerve distribution distal to the wrist EC: 1) Prior carpal tunnel release, 2) Neurologic diseases such as ipsilateral stroke, multiple sclerosis, paresthesia limited to digits four and five, or cervical myelopathy, 3) Patients referred for CTS evaluation who did not have median distribution paresthesia.
**Raudino 2000** [28]	Mean = 48.9, SD = 13.9 years, Range = 19–82 / NR/ consecutive	NR/ Mean duration = 26.9 + 38.1 months, Range = 1–180 months	Referred for electrodiagnostic evaluation	IC: According to the diagnostic criteria of the American Academy of Neurology, all patients were complaining of discomfort, paresthesia or weakness in the territory of the median nerve occurring especially at night or after repetitive actions and relieved by changes in posture or shaking hand. EC: Metabolic diseases, radiculopathies or polyneuropathies were exclusion criteria. If adequate, other electrophysiologic studies or needle electromyography were performed in order to exclude concomitant pathologies.
**Sartorio et al. 2017** [10]	Severe CTS: Mean = 56.1, SD = 11.7; Moderate: Mean = 54.51, SD = 8.21; Mild: Mean = 51.6, SD = 7.7; Negative: Mean = 49.1, SD = 8.5/ Severe: 80% W; Moderate: 77.1% W; Mild: 88.2% W; Negative: 55.6% W/ consecutive	Severe (*n* = 10), Moderate (*n* = 35) Mild (*n* = 17) Negative (*n* = 18) / NR	In the period between January and July 2015 at the Laboratory of Ergonomics and Evaluation of Musculoskeletal Disorders of the Clinical Scientific Institutes Maugeri.	IC: All patients between 40 and 70 years of age. EC: 1) fractures or surgical interventions in the upper limb; 2) cervical whiplash in the last three months; 3) amputations of the 1st, 2nd and 3rd fingertips; 4) pregnancy; 5) polyneuropathies or relapse of STC; 6) hypo/hyperthyroidism; 7) outcomes of treatment with neurotoxic drugs (antineoplastic).
**Szabo et al. 1999** [29]	CTS: range = 20–73, Non-CTS: range = 28–72; Healthy: range = 18–59 / CTS: 38 W, 12 M Non-CTS: 40 W, 10 M Healthy: 3 W, 17 M/ consecutive	NR/ Group 1: diagnosed CTS: 2 months to 20 years Group 2: other hand pathologies: 2 weeks to 15 years Group 3: good general health and lack of upper extremity pathology	Consecutive patients evaluated and treated at an institution for hand, wrist, and forearm problems between 1993 and 1996 Group 3: healthy volunteers recruited from the general population and included university students, medical center employees, and their friends and relatives.	IC: Group 1: a clinical history of numbness and tingling in the median nerve distribution and/or night pain relieved by shaking of the hand; results of physical examination, including sensibility and provocative examinations, consistent with carpal tunnel syndrome; and relief of symptoms after carpal tunnel release Group 2: Diagnoses included epicondylitis, de Quervain’s and other tendinosis, radiculopathy, and hand pain of unknown etiology. Group 3: good general health and lack of UE pathology and symptoms. EC: NR
**Yildirim & Gunduz 2015** [6]	Mean = 49.09, SD = 10.5, Range = 20–72 / 8.1% M/ consecutive	Mild CTS = 19, moderate CTS = 18, Severe CTS = 12 / NR	Patients who applied to the outpatient clinic of a university with symptoms suggesting CTS were assessed retrospectively	IC: NR EC: 1) Presence of a neurologic disease; 2) Prior nerve injuries, trauma, or a surgical procedure in the upper extremities; 3) Thenar atrophy; pregnancy; or acute/ subacute cervical radiculopathy; 4) Patients who had clinical or electrophysiological findings suggesting other pathologies, such as polyneuropathy, ulnar, and/or radial neuropathy.

List of Abbreviations: *IC* inclusion criteria, *EC* exclusion criteria, *NR* not reported, *W* women, *M* men, *CTS* carpal tunnel syndrome, *SD* standard deviation

### Risk of Bias and applicability concerns of the included studies

All of the studies had low risk of bias rating in the patient selection domain of the QUADAS-2 and enrolled a consecutive sample of participants, avoided a case-control design and inappropriate exclusions. Six studies had unclear risk of bias ratings in the index test domain. It was unclear if the index tests results were interpreted without the knowledge of the results of the reference standard. Three studies had high, seven studies had unclear, and six studies had low risk of bias in the reference standard domain. The main reason for low ratings was the lack of blinding of the person performing the reference standard test. Eleven studies had unclear ratings on the flow and timing domain, because there was no mention of the appropriate interval between index and reference standard tests administration.

**Fig. 2:**
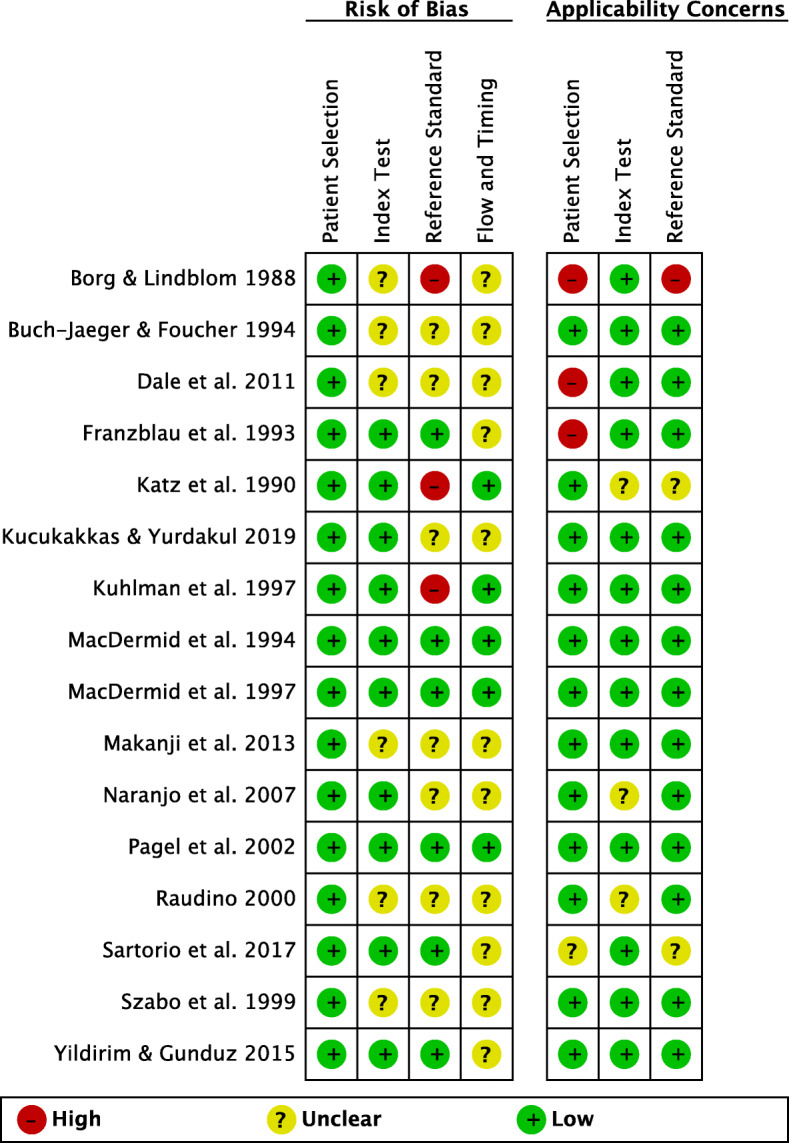
Risk of bias and applicability concerns of the included studies, using QUADAS-2 tool

**Fig. 3:**
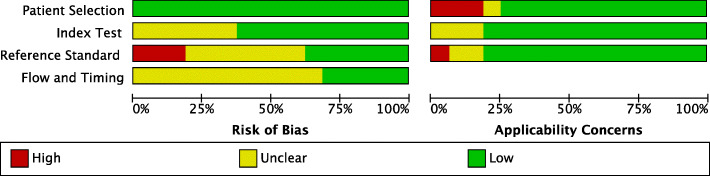
The proportion of included studies with low, high, or unclear risk of bias and concerns regarding the applicability, using QUADAS-2 tool

Regarding the applicability concerns of the included studies, nine studies had low concerns, four had unclear, and three studies had high concerns. In the patient selection domain, three studies had high, one study had unclear, and eleven studies had low applicability concerns. In the index test domain, only three studies had unclear concerns and the rest of the studies (thirteen studies) had no concerns regarding applicability. Lastly, in the reference standard domain, one study had high concerns, two studies had unclear concerns, and thirteen studies had no concerns regarding applicability. The visual demonstration of the risk of bias and applicability concerns of the included studies is presented in Figs. 2 and 3.

### Diagnostic accuracy of sensory tests for CTS diagnosis

The diagnostic accuracies of the SWMFs, two-point discrimination, vibrometry, hypoesthesia, tactile thresholds, Von Frey hairs, graphesthesia, and warm and cold thresholds were assessed in the included studies. See Tables 4 and 5 for detailed results.

**Table 4:** Diagnostic Accuracy of the Semmes-Weinstein monofilament test for CTS diagnosis

Study (Authors, year)	Sensitivity (%)	Specificity (%)	PPV (%)	NPV (%)	+LR	-LR
**Buch-Jaeger & Foucher 1994** [21]	59	59	65	46	1.43*	0.69*
**Dale et al. 2011** [8]	**RS#1** L = 34.6 R = 57.9	**RS#1** L = 74.1 R = 69.8	**RS#1** L = 3.1 R = 6.4	**RS#1** L = 97.9 R = 97.9	**RS#1** L = 1.3 (0.78–2.27) R = 1.9 (1.44–2.53)	**RS#1** L = 0.9 (0.67–1.17) R = 0.6 (0.41–0.88)
	**RS#2** L = 35.7 R = 45.2	**RS#2** L = 76.8 R = 74.8	**RS#2** L = 33.2 R = 41.9	**RS#2** L = 78.7 R = 77.3	**RS#2** L = 1.5 (1.25–1.83) R = 1.8 (1.51–2.07)	**RS#2** L = 0.8 (0.76–0.92) R = 0.7 (0.66–0.82)
	**RS#3** L = 54.5 R = 66.7	**RS#3** L = 74.0 R = 69.9	**RS#3** L = 2.1 R = 4.9	**RS#3** L = 99.4 R = 98.9	**RS#3** L = 2.1 (1.21–3.61) R = 2.2 (1.64–2.96)	**RS#3** L = 0.6 (0.32–1.17) R = 0.5 (0.27–0.84)
**MacDermid et al. 1994** [25]	**SWMF > 2.83** Tester 1 = 97 Tester 2 = 97	**SWMF > 2.83** Tester 1 = 23 Tester 2 = 9	NR	NR	**SWMF > 2.83** Tester 1 = 1.25* Tester 2 = 1.06*	**SWMF > 2.83** Tester 1 = 0.13* Tester 2 = 0.33*
	**SWMF > 2.83 & > D5** Tester 1 = 86 Tester 2 = 85	**SWMF > 2.83 & > D5** Tester 1 = 60 Tester 2 = 32			**SWMF > 2.83 & > D5** Tester 1 = 2.15* Tester 2 = 1.25*	**SWMF > 2.83 & > D5** Tester 1 = 0.23* Tester 2 = 0.46*
	**SWMF > 3.22** Tester 1 = 79 Tester 2 = 96	**SWMF > 3.22** Tester 1 = 64 Tester 2 = 34			**SWMF > 3.22** Tester 1 = 2.19* Tester 2 = 1.45*	**SWMF > 3.22** Tester 1 = 0.32* Tester 2 = 0.18*
	**SWMF > 3.22 & > D5** Tester 1 = 70 Tester 2 = 72	**SWMF > 3.22 & > D5** Tester 1 = 70 Tester 2 = 47			**SWMF > 3.22 & > D5** Tester 1 = 2.33* Tester 2 = 1.35*	**SWMF > 3.22 & > D5** Tester 1 = 0.42* Tester 2 = 0.59*
**MacDermid et al. 1997** [26]	Tester 1 = 86 Tester 2 = 85	Tester 1 = 60 Tester 2 = 32	NR	NR	Tester 1 = 2.15* Tester 2 = 1.25*	Tester 1 = 0.23* Tester 2 = 0.46*
**Pagel et al. 2002** [27]	**SWM > 2.83** 98	**SWM > 2.83** 15	**SWM > 2.83** 56	**SWM > 2.83** 88	**SWM > 2.83** 1.15*	**SWM > 2.83** 0.13*
	**SWMF > 2.83 & > D5** 13	**SWMF > 2.83 & > D5** 88	**SWMF > 2.83 & > D5** 53	**SWMF > 2.83 & > D5** 47	**SWMF > 2.83 & > D5** 1.08*	**SWMF > 2.83 & > D5** 0.98*
**Szabo et al. 1999** [29]	**Neutral:** 65 (95%CI 52–75)	**Neutral:** 42 (95%CI 30–52)	**Neutral:** 1%*P* = 1 5%*P* = 6 10%*P* = 11 15%*P* = 17 20%*P* = 22	**Neutral:** 1%*P* = 99 5%*P* = 96 10%*P* = 92 15%*P* = 87 20%*P* = 83	**Neutral:** 1.12*	**Neutral:** 0.83*
	**Phalen’s:** 83 (95%CI 69–88)	**Phalen’s:** 44 (95%CI 32–55)	**Phalen’s:** 1%*P* = 1 5%*P* = 7 10%*P* = 14 15%*P* = 21 20%*P* = 27	**Phalen’s:** 1%*P* = 99 5%*P* = 98 10%*P* = 96 15%*P* = 94 20%*P* = 91	**Phalen’s:** 1.48*	**Phalen’s:** 0.38*
**Yildirim & Gunduz 2015** [6]	**SWMF > 2.83** 98	**SWMF > 2.83** 17	**SWMF > 2.83** 44	**SWMF > 2.83** 93	**SWMF > 2.83** 1.18*	**SWMF > 2.83** 0.12*
	**SWMF > 3.22** 49	**SWMF > 3.22** 93	**SWMF > 3.22** 83	**SWMF > 3.22** 74	**SWMF > 3.22** 7*	**SWMF > 3.22** 0.55*

*PPV* Positive Predictive Value, *NPV* Negative Predictive Value, *+LR* Positive Likelihood Ratio, *−LR* Negative Likelihood Ratio, *CI* confidence interval, *NR* not reported, *RS* reference standard, *SWMFs* Semmes-Weinstein monofilaments

In Dale et al. ‘s study, the first reference standard (RS#1) was the modified Katz hand diagram, the second reference standard (RS#2) was NCS, and the third reference standard (RS#3) was a consensus criteria of CTS case definition; MacDermid et al. ‘s study (1997) reported the SWMFs test results based on four different decision rules, which we have reported separately in the table; In Szabo’s study, the positive and negative predicted values were calculated based on five hypothetical CTS prevalence (1, 5, 10, 15, and 20%) and two testing positions (neutral, Phalen’s) which we reported separately in the table; * values calculated by the authors of this study

**Table 5:** Diagnostic Accuracy of Vibrometry for CTS diagnosis

Study (Authors, year)	Sensitivity (%)	Specificity (%)	PPV (%)	NPV (%)	+LR	-LR
**Borg & Lindblom 1988** [20]	52	NR	NR	NR	NR	NR
**Buch-Jaeger & Foucher 1994** [21]	26	77	60	44	1.13*	0.96*
**Franzblau et al. 1993** [22]	**RS#1** 3	**RS#1** 91	**RS#1** 6	**RS#1** 84	**RS#1** 0.33*	**RS#1** 1.06*
	**RS#2** 5	**RS#2** 92	**RS#2** 10	**RS#2** 85	**RS#2** 0.62*	**RS#2** 1.03*
	**RS#3** 11	**RS#3** 93	**RS#3** 21	**RS#3** 86	**RS#3** 1.57*	**RS#3** 0.95*
**MacDermid et al. 1997** [26]	Tester 1 = 77 Tester 2 = 77	Tester 1 = 80 Tester 2 = 72	NR	NR	Tester 1 = 3.85* Tester 2 = 2.75*	Tester 1 = 0.28* Tester 2 = 0.31*

*PPV* Positive Predictive Value, *NPV* Negative Predictive Value, *+LR* Positive Likelihood Ratio, *−LR* Negative Likelihood Ratio, *CI* confidence interval, *NR* not reported, *RS* reference standard

In Franzblau et al. ‘s study, the first reference standard was electrodiagnosis, the second reference standard was electrodiagnosis and symptoms consistent with CTS, and the third reference standard was physical examination findings and symptoms consistent with CTS;

* values calculated by the authors of this study

Semmes-Weinstein monofilaments (SWMFs) test was assessed in seven of the included studies [6, 8, 21, 25–27, 29]. The reported sensitivities and the specificities ranged from 13 to 98%, and from 9 to 93%, respectively [6, 8, 21, 25–27, 29]. The authors of this SR calculated +LR and -LR, which ranged from 1.6 to 7, and from 0.98 to 0.12, respectively. Different decision rules were tested in the studies, which resulted in different diagnostic accuracies, and are summarized in Table 4. In the study by Szabo et al. 1999, SWMFs was performed in two positions, neutral and Phalen’s position (90 degrees of wrist flexion) [29]. The results from this study indicated a better diagnostic accuracy for SWMFs test, when done with wrist flexion (Sn = 83%, Sp = 44%, +LR = 1.48, −LR = 0.38) [29]. Furthermore, Szabo et al., calculated the PPV and NPV based on five hypothetical CTS prevalence, ranging from 1 to 20% [29], with the details of this analysis being summarized in Table 4.

Two-point discrimination test was assessed in four studies [7, 20, 21, 23]. In the study by Borg & Lindblom, only the Sn was calculated, which was 30% [20]. In the other three studies, the Sn was 6, 32, and 63%, the Sp was 98, 81, and 85%, the +LR was 3, 1.68, and 4.2, and the -LR was 0.95 and 0.84, and 0.43.^7,20,217^ In the study by Katz et al. 1990, PPV and NPV were calculated based on two CTS prevalence [23]. In a sample with 40% CTS prevalence (sample 1), the PPV was 54%, with a 95% CI ranging from 37 to 70, and the NPV was 63% (95%CI 58 to 68) [23]. In sample 2 with a CTS prevalence of 15%, the PPV was 23% and the NPV was 87% [23].

Vibrometry was assessed in four studies [20–22, 26]. In the study by Borg et al. 1988 [20], only Sn was calculated for vibrometry testing, which was 52%. Franzblau et al., incorporated three different reference standards, which were NCS; NCS + symptoms consistent with CTS; and physical examination findings and symptoms consistent with CTS [22]. The highest diagnostic accuracy values occurred when taking physical examination findings as the reference standard (Sn = 11%, Sp = 93%, +LR = 1.57, −LR = 0.95) [22]. In the study by MacDermid et al. 1997, two testers performed the vibrometry [26], which resulted in different diagnostic accuracies as summarised in Table 5.

Hypoesthesia was another form of sensory testing for CTS diagnosis assessed in our included studies. In a study by Raudino (2000), only the Sn was calculated, which was 32% [28]. In another study, the following diagnostic accuracy properties were reported: Sn = 51%, Sp = 85%, PPV = 85%, NPV = 51%, +LR = 3.4, and -LR = 0.57 [24].

Lastly, Tactile thresholds, Von Frey hairs, graphesthesia, and warm and cold thresholds were only assessed in one study [20]. In this study by Borg & Lindblom, only the Sn was calculated, which was 52% for tactile thresholds, 52% for Von Frey hairs test, 24% for graphesthesia, and 15% for warm and cold thresholds [20]. Borg & Lindblom assessed the diagnostic accuracy of six sensory tests, which were vibrometry, two-point discrimination, tactile thresholds, Von Frey hairs, graphesthesia, and warm and cold thresholds. They called this combination, quantitative sensory testing (QST), and it had a Sn of 82% [20].

### Diagnostic accuracy of motor tests for CTS diagnosis

The motor tests assessed in the included studies were thumb abduction weakness, thenar atrophy, hand grip strength, pinch grip strength, and functional dexterity tests. Each test is summarized below, and detailed information can be found in Table 6.

**Table 6:** Diagnostic Accuracy of Hand Grip Strength, Pinch Grip Strength, Thumb Abduction Weakness, Thenar Atrophy, and Functional dexterity tests for CTS diagnosis

Study (Authors, year)	Sensitivity (%)	Specificity (%)	PPV (%)	NPV (%)	+LR	-LR
**Hand (palmar) grip strength**						
**Franzblau et al. 1993** [22]	**RS#1** 10	**RS#1** 90	**RS#1** 15	**RS#1** 85	**RS#1** 1*	**RS#1** 1*
	**RS#2** 10	**RS#2** 90	**RS#2** 15	**RS#2** 85	**RS#2** 1*	**RS#2** 1*
	**RS#3** 32	**RS#3** 94	**RS#3** 47	**RS#3** 89	**RS#3** 5.33*	**RS#3** 0.72*
**Szabo et al. 1999** [29]	48 (95% CI 26–70)	30 (95% CI 14–46)	1%*P* = 1 5%*P* = 3 10%*P* = 7 15%*P* = 11 20%*P* = 15	1%*P* = 98 5%*P* = 92 10%*P* = 84 15%*P* = 77 20%*P* = 70	0.68*	1.73*
**Pinch grip strength**						
**Franzblau et al. 1993** [22]	**RS#1** 10	**RS#1** 93	**RS#1** 20	**RS#1** 85	**RS#1** 1.42*	**RS#1** 0.96*
	**RS#2** 20	**RS#2** 95	**RS#2** 39	**RS#2** 87	**RS#2** 4*	**RS#2** 0.84*
	**RS#3** 21	**RS#3** 95	**RS#3** 41	**RS#3** 87	**RS#3** 4.2*	**RS#3** 0.83*
**MacDermid et al. 1997** [26]	Tester 1 = 72 Tester 2 = 70	Tester 1 = 88 Tester 2 = 78	NR	NR	Tester 1 = 6.00* Tester 2 = 3.18*	Tester 1 = 0.31* Tester 2 = 0.38*
**Thumb abduction weakness**						
**Kuhlman et al. 1997** [24]	66	66	76	54	1.94*	0.51*
**Makanji et al. 2013** [11]	37	73	80	28	1.37*	0.86*
**Raudino 2000** [28]	12.1	NR	NR	NR	NR	NR
**Thenar atrophy**						
**Kucukakkas & Yurdakul 2019** [7]	22 (95% CI 17–28)	100 (95% CI 98–100)	100	57 (95% CI 56–59)	Infinite*	0.78*
**Makanji et al. 2013** [11]	18	96	92	29	4.5*	0.85*
**Naranjo et al. 2007** [9]	5.5	100	NR	NR	Infinite	0.95
**Functional dexterity test**						
**Sartorio et al. 2017** [10]	84 (95% CI 72–90)	64 (95% CI 41–82)	NR	NR	2.37 (95% CI, 1.23–4.55)	0.25 (95% CI, 0.13–0.49)

*PPV* Positive Predictive Value, *NPV* Negative Predictive Value, *+LR* Positive Likelihood Ratio, *−LR* Negative Likelihood Ratio, *CI* confidence Interval, *NR* Not Reported, *RS* reference standard

In Franzblau et al. ‘s study, the first reference standard was electrodiagnosis, the second reference standard was electrodiagnosis and symptoms consistent with CTS, and the third reference standard was physical examination findings and symptoms consistent with CTS;

In Szabo’s study, the positive and negative predicted values were calculated based on five hypothetical CTS prevalence, which were 1, 5, 10, 15, and 20% which we reported separately in the table;

* values calculated by the authors of this study

Thumb abduction weakness was assessed in three studies [24, 28, 30]. The reported sensitives and specificities from these studies ranged from 12.1 to 66%, and from 66 to 73%, respectively [24, 28, 30]. As calculated by the authors of this study, the +LR were 1.37, and 1.94, and the -LR were 0.51 and 0.86 for thumb abduction weakness testing [24, 30]. We could only obtain the values for sensitivity from Raudino 2000 study [28].

Thenar atrophy was assessed by three studies [7, 9, 11]. The Sn of the thenar atrophy test was minimal, with values ranging from 5.5 to 22%, but it was a highly specific test, with Sp ranging from 96 to 100% [9, 11].

Hand grip strength was assessed in two studies. In Franzblau et al. ‘s study [22], hand grip strength was compared to three different reference standards: 1) electrodiagnosis, 2) electrodiagnosis and symptoms consistent with CTS, and 3) physical examination findings and symptoms consistent with CTS [22]. The highest diagnostic accuracy results came from taking physical examination findings and symptoms consistent with CTS as the reference standard, which yielded a Sn of 32% and a Sp of 94% [22]. As calculated by the authors of this study, hand grip strength testing had a + LR of 5.33, and a -LR of 0.72. In addition, Szabo et al. 1999 found that hand grip strength had the following diagnostic accuracy: Sn = 48 (95% CI 26–70), Sp = 30 (95% CI 14–46) [29]. Positive and negative predictive values were calculated using five hypothetical CTS prevalence, which are summarized in Table 6. In general, the lowest CTS prevalence (1%) resulted in the worst PPV (1%) and the best NPV (98%) [29].

Pinch grip strength was assessed in two studies. In a study by MacDermid et al., two testers performed the pinch grip strength testing and identified Sn of 72 and 70% for testers 1, and 2, respectively [26]. The Sp values were 88% for tester 1 and 78%, for tester 2 [26]. According to Franzblau et al., when taking physical examination findings and symptoms consistent with CTS as the reference standard, pinch grip strength had a Sn 21%, and Sp 95% [22].

Functional dexterity test was only assessed by Sartorio et al. 2017, and was found to have Sn of 84% (95% CI 72–90%), Sp of 64% (95% CI 41–82%), +LR of 2.37 (95% CI, 1.23–4.55), and -LR of 0.25 (95% CI, 0.13–0.49) [10].

### Reference standards for CTS diagnosis

Out of the 16 included studies, 11 studies had nerve conduction studies (NCS) as their reference standard. These studies had different criteria for positive test results, which are summarized in Appendix C. In the remaining five studies, the following reference standards were considered. Borg & Lindblom [20] (1988) used a combined battery of tests as the reference standard. This combined battery of tests included formal CTS screening, the neurological examination and the electrophysiological testing. No further information was provided by the authors. Dale et al. 2012, had three different reference standards: 1)Modified Katz hand diagram and people suspected with CTS were categorized as having ‘classic, probable, possible, or unlikely’ CTS; 2) NCS; 3)A consensus criteria for CTS case definition, requiring having classic or probable CTS rating on the modified Katz hand diagram, and abnormal median nerve conduction testing [8]. MacDermid et al. used a clinical diagnosis by a specialist hand surgeon combined with NCS as their reference standard [25, 26]. Finally, Franzblau et al. 1993, had three different reference standards, which were 1)NCS; 2)NCS + surveillance symptom definitions for CTS; 3)Physical examination + surveillance symptom definitions for CTS [22].

## Discussion

This study synthesized sixteen clinical studies reporting on thirteen different sensory and motor tests. Among these tests, none had consistent evidence for high diagnostic accuracy. These results suggest clinicians should not rely on the results of one single sensory or motor test for CTS diagnosis, instead using a combination of several of sensory and motor tests, or other combinations of tests from different AAOS categories to rule in/rule out CTS.

In this SR, we found the most specific tests for CTS diagnosis were the hand (palmar) grip strength test [22] (Sp of 94%), pinch grip strength, (Sp from 78% [26] to 95% [22]), thenar atrophy (Sp from 96 to 100%) [7, 9, 30], and 2PD (Sp from 81 to 98%) [7, 21, 23]. Tests with high Sp can detect true negative cases with a great precision and have a low false positive outcome [18]. This feature can assist clinicians in differentiating between CTS and non-CTS cases. Of the included sensory and motor tests, the most sensitive for CTS diagnosis was the SWMF test, using the 3.22 monofilament size in any radial finger as the normal threshold, with Sn values ranging from 49% [6] to 96% [26]. Tests with high sensitivity have low false negative results, which is an important factor for screening purposes [31]. In other words, when the objective of a clinician is to screen people with suspected CTS, they should use a highly sensitive test (low specificity values are tolerable); therefore, the SWMF is potentially a useful screening tool.

Our results confirm the findings of a recent clinical practice guideline by the Academy of Hand and Upper Extremity Physical Therapy and the Academy of Orthopedic Physical Therapy of the American Physical Therapy Association [32]. This guideline recommended using the SMWFs test with either 3.22 or 2.83 as the normal threshold for mild to moderate CTS cases, and for more severe CTS cases, a 3.22 threshold should be considered. Compared to the previous SR on this topic by MacDermid and Wessel in 2004 [5], in this updated SR we mainly focused on a sample with no healthy controls, and this was the main difference of the two SRs. Moreover, MacDermid and Wessel concluded that the most specific (but not sensitive) tests for CTS were the 2PD and testing of thumb abduction weakness [5]. We did find the 2PD as one of the most specific tests, however, the palmar and pinch grip strength tests, and the atrophy of the thenar muscles proved more specific than the thumb abduction weakness test.

Only two studies reported the prevalence of CTS in the underlying population where they sampled their participants from [23, 29]. Prevalence is important when considering applying the results since the pretest probability is determined by the prevalence [18]. Settings with higher prevalence of CTS, such as electrodiagnosis labs and hand therapy clinics, likely have higher pre-test probability of CTS as compared to other screening contexts such as preemployment screening, where the prevalence would be expected to be very low. Except for two studies [8, 22], all of the included studies recruited their participants from neurophysiology/electrodiagnosis and hand clinics. To overcome the effect of CTS pretest probability, we ensured likelihood ratios were reported in this SR. Likelihood ratios report diagnostic accuracy independent from the prevalence of a condition in a given sample, and it is suggested that clinicians consider likelihood ratios in their clinical diagnosis decision making [18].

Administration methods of the sensory and motor tests for CTS diagnosis were very diverse across the included studies. For instance, the four studies assessing the diagnostic accuracy of vibrometry had four different methods in testing and different decision rules for positive test results. The same principle applies to the hand grip strength, hypoesthesia, pinch grip strength, and SWMFs tests. We advise clinicians and researchers should carefully consider their ability to replicate test methods (as reported in Table 2) when deciding on selecting a sensory or motor test to rule in/out CTS.

We did not exclude studies based on the choice for reference standard. Due to the lack of a gold standard for CTS diagnosis [4], and the nature of CTS as a clinical syndrome, there is no universal agreement on a reference standard. The most commonly used reference standard in the included studies was NCS. While some might consider NCS as the most definitive reference standard, it can have false positive and negative results [4]. That is, there can be abnormal results in patients who have no symptoms, and patients with persistent symptoms without positive NCS can show benefit following carpal tunnel release. Similar to our previous SR of the diagnostic accuracy of scales, questionnaires and hand symptom diagrams [12], the highest sensitivities and specificities occurred when taking other clinical tests and history as the reference standard [8, 22, 25, 26]. For instance, in the study done by Dale et al. 2011, among the three reference standards used, the highest diagnostic accuracy values occurred when taking Katz and Stirrat’s hand symptom diagram as the reference standard [8].

### Study limitations and future directions

A limitation of the current study was that we did not conduct a meta-analysis. Due to the heterogeneity in the tests methods, reference standards, and decision rules for positive tests thresholds, meta-analysis was precluded, and we reported the results narratively. A second limitation that we would like to acknowledge is the possibility of a publication bias, because we only included published literature, not the gray literature. Our choice of only including published literature is justifiable by the argument that we intended to produce a synthesis of the available peer-reviewed evidence-based literature. As with any other review, we might have missed some studies. Although we designed the search strategy in consultation with a health science research librarian, it is possible that we did not capture all of the available evidence.

We recommend future studies produce evidence with the highest quality and the lowest risk of bias by adhering strictly to the established guidelines. Moreover, there is a great need for studies assessing the clinical triangulation process of combining several categories of clinical diagnostic tests.

## Conclusion

The evidence reported in this study was obtained mostly from studies at risk of bias. Among the included studies none of the sensory or motor tests had consistently high diagnostic accuracy properties reported by high quality evidence. Confirming the value of a single sensory or motor test for CTS diagnosis is pending future robust research. From the evidence available at present, none of these methods appear promising in helping to make a definitive diagnosis in the individual patient (though they are useful in demonstrating that both sensory and motor function are impaired by CTS when used in cohorts of patients in research studies).

## Data Availability

All data generated or analysed during this study are included in this published article [and its supplementary information files].

## Appendix A

### Search strategy

#### OVID Medline Search Strategy

1. Carpal Tunnel Syndrome/
2. Carpal Tunnel Syndrome.mp.
3. Carpal Tunnel Syndrome/ or Nerve Compression Syndromes/ or Median Neuropathy/
4. Carpal Tunnel Syndrome/di [Diagnosis]
5. Median Neuropathy/di [Diagnosis]
6. median nerve entrapment*.mp. [mp = title, abstract, original title, name of substance word, subject heading word, floating sub-heading word, keyword heading word, protocol supplementary concept word, rare disease supplementary concept word, unique identifier, synonyms]
7. compression neuropathy.mp. [mp = title, abstract, original title, name of substance word, subject heading word, floating sub-heading word, keyword heading word, protocol supplementary concept word, rare disease supplementary concept word, unique identifier, synonyms]
8. Nerve Compression Syndromes/
9. cts.mp. [mp = title, abstract, original title, name of substance word, subject heading word, floating sub-heading word, keyword heading word, protocol supplementary concept word, rare disease supplementary concept word, unique identifier, synonyms]
10. syndrome, carpal tunnel.mp. [mp = title, abstract, original title, name of substance word, subject heading word, floating sub-heading word, keyword heading word, protocol supplementary concept word, rare disease supplementary concept word, unique identifier, synonyms]
11. 1 or 2 or 3 or 4 or 5 or 6 or 7 or 8 or 9 or 10
12. diagnostic test*.mp. [mp = title, abstract, original title, name of substance word, subject heading word, floating sub-heading word, keyword heading word, protocol supplementary concept word, rare disease supplementary concept word, unique identifier, synonyms]
13. clinical test*.mp.
14. diagnostic accuracy.mp. [mp = title, abstract, original title, name of substance word, subject heading word, floating sub-heading word, keyword heading word, protocol supplementary concept word, rare disease supplementary concept word, unique identifier, synonyms]
15. “Sensitivity and Specificity”/
16. sensitivity.mp. [mp = title, abstract, original title, name of substance word, subject heading word, floating sub-heading word, keyword heading word, protocol supplementary concept word, rare disease supplementary concept word, unique identifier, synonyms]
17. specificity.mp. [mp = title, abstract, original title, name of substance word, subject heading word, floating sub-heading word, keyword heading word, protocol supplementary concept word, rare disease supplementary concept word, unique identifier, synonyms]
18. roc curve.mp. [mp = title, abstract, original title, name of substance word, subject heading word, floating sub-heading word, keyword heading word, protocol supplementary concept word, rare disease supplementary concept word, unique identifier, synonyms]
19. 12 or 13 or 14 or 15 or 16 or 17 or 18
20. 11 and 19
21. (“Symptom diagram” or “hand diagram” or “Flick sign” or “Provocative Test*” or “Phalen’s test” or “phalen test” or “wrist flexion test” or “wrist extension test” or “reverse Phalen test” or “carpal compression test” or “Durkan’s test” or “Tinel’s sign” or “Tourniquet test” or “Gilliat test” or “Sensory test*” or “Motor Test*” or “Touchor vibration threshold” or “Current perception threshold” or “Two-point discrimination Semmes-Weinstein Monofilament Test” or “Thenar weakness” or “Thumb Abduction Weakness” or “thenar atrophy” or “Abductor Pollicis Brevis Manual Muscle Testing” or “CTS-Relief Maneuver” or “CTS-RM” or “Pin Prick Sensory Deficit” or “ULNT Criterion C” or “upper limb neurodynamic test Tethered median nerve stress test” or “Luthy’s sign” or “luthy sign” or “scratch collapse test” or “Pinwheel” or “CTS-6 evaluation tool” or “The Alderson-McGall hand function questionnaire” or “Hand elevation test” or “Katz and Stirrat hand diagram” or “katz hand diagram” or “Purdue Pegboard Test” or “Levine’s Self-Assessment Questionnaire” or “Dellon-modified Moberg pick-up test” or “Self-administered diagram” or “web-based questionnaire” “Kamath and Stothard questionnaire” or “Lo Carpal Tunnel Questionnaire” or “scratch-collapse test” or “hyperextension test” or “Flinn Performance Screening Tool” or “FPST”).mp. [mp = title, abstract, original title, name of substance word, subject heading word, floating sub-heading word, keyword heading word, protocol supplementary concept word, rare disease supplementary concept word, unique identifier, synonyms]
22. 20 and 21

#### OVID EMBASE Search Strategy

1. carpal tunnel syndrome.mp. [mp = title, abstract, heading word, drug trade name, original title, device manufacturer, drug manufacturer, device trade name, keyword, floating subheading word, candidate term word]
2. median neuropath*.mp. [mp = title, abstract, heading word, drug trade name, original title, device manufacturer, drug manufacturer, device trade name, keyword, floating subheading word, candidate term word]
3. median nerve entrapment*.mp. [mp = title, abstract, heading word, drug trade name, original title, device manufacturer, drug manufacturer, device trade name, keyword, floating subheading word, candidate term word]
4. compression neuropath*.mp. [mp = title, abstract, heading word, drug trade name, original title, device manufacturer, drug manufacturer, device trade name, keyword, floating subheading word, candidate term word]
5. entrapment neuropath*.mp. [mp = title, abstract, heading word, drug trade name, original title, device manufacturer, drug manufacturer, device trade name, keyword, floating subheading word, candidate term word]
6. carpal canal syndrome.mp. [mp = title, abstract, heading word, drug trade name, original title, device manufacturer, drug manufacturer, device trade name, keyword, floating subheading word, candidate term word]
7. carpal tunnel compression*.mp. [mp = title, abstract, heading word, drug trade name, original title, device manufacturer, drug manufacturer, device trade name, keyword, floating subheading word, candidate term word]
8. “neuropathy, median “.mp. [mp = title, abstract, heading word, drug trade name, original title, device manufacturer, drug manufacturer, device trade name, keyword, floating subheading word, candidate term word]
9. “syndrome,carpal tunnel”.mp. [mp = title, abstract, heading word, drug trade name, original title, device manufacturer, drug manufacturer, device trade name, keyword, floating subheading word, candidate term word]
10. carpal tunnel syndrome/
11. 1 or 2 or 3 or 4 or 5 or 6 or 7 or 8 or 9 or 10
12. clinical test*.mp.
13. “sensitivity and specificity”/
14. receiver operating characteristic/
15. differential diagnosis.mp. [mp = title, abstract, heading word, drug trade name, original title, device manufacturer, drug manufacturer, device trade name, keyword, floating subheading word, candidate term word]
16. “diagnostic test*”.mp. [mp = title, abstract, heading word, drug trade name, original title, device manufacturer, drug manufacturer, device trade name, keyword, floating subheading word, candidate term word]
17. (“sensitivity” or “specificity”).mp. [mp = title, abstract, heading word, drug trade name, original title, device manufacturer, drug manufacturer, device trade name, keyword, floating subheading word, candidate term word]
18. “ROC curve” .mp. [mp = title, abstract, heading word, drug trade name, original title, device manufacturer, drug manufacturer, device trade name, keyword, floating subheading word, candidate term word]
19. diagnostic accuracy/ or diagnostic test accuracy study/ or differential diagnosis/ or physical examination/
20. 12 or 13 or 14 or 15 or 16 or 17 or 18 or 19
21. 11 and 20
22. (“Symptom diagram” or “hand diagram” or “Flick sign” or “Provocative Test*” or “Phalen’s test” or “phalen test” or “wrist flexion test” or “wrist extension test” or “reverse Phalen test” or “carpal compression test” or “Durkan’s test” or “Tinel’s sign” or “Tourniquet test” or “Gilliat test” or “Sensory test*” or “Motor Test*” or “Touchor vibration threshold” or “Current perception threshold” or “Two-point discrimination Semmes-Weinstein Monofilament Test” or “Thenar weakness” or “Thumb Abduction Weakness” or “thenar atrophy” or “Abductor Pollicis Brevis Manual Muscle Testing” or “CTS-Relief Maneuver” or “CTS-RM” or “Pin Prick Sensory Deficit” or “ULNT Criterion C” or “upper limb neurodynamic test Tethered median nerve stress test” or “Luthy’s sign” or “luthy sign” or “scratch collapse test” or “Pinwheel” or “CTS-6 evaluation tool” or “The Alderson-McGall hand function questionnaire” or “Hand elevation test” or “Katz and Stirrat hand diagram” or “katz hand diagram” or “Purdue Pegboard Test” or “Levine’s Self-Assessment Questionnaire” or “Dellon-modified Moberg pick-up test” or “Self-administered diagram” or “web-based questionnaire” or “scratch-collapse test” or “hyperextension test” or “Kamath and Stothard questionnaire” or “Lo Carpal Tunnel Questionnaire” or “Flinn Performance Screening Tool” or “FPST”).mp. [mp = title, abstract, heading word, drug trade name, original title, device manufacturer, drug manufacturer, device trade name, keyword, floating subheading word, candidate term word]
23. 21 and 22

#### CINAHL search strategy

1. (MH “Carpal Tunnel Syndrome”)
2. “median neuropath*”
3. “median nerve entrapment*”
4. “compression neuropath*”
5. “entrapment neuropath*”
6. S1 OR S2 OR S3 OR S4 OR S5
7. “diagnosis or assessment”
8. “diagnosis”
9. “diagnostic”
10. (MH “Diagnosis”) OR (MH “Diagnosis, Neurologic”) OR (MH “Diagnosis, Musculoskeletal”) OR (MH “Exercise Test”) OR (MH “Functional Assessment”) OR (MH “Patient Assessment”) OR (MH “Patient History Taking”) OR (MH “Physical Examination”) OR (MH “Sensitivity and Specificity”)
11. (MH “Diagnosis, Musculoskeletal”) OR (MH “Diagnosis, Neurologic”) OR (MH “Functional Assessment”) OR (MH “Patient Assessment”) OR (MH “Patient History Taking”) OR (MH “Physical Examination”) OR (MH “Sensitivity and Specificity”) OR (MH “Skin Tests”)
12. (MH “Sensitivity and Specificity”) OR “sensitivity and specificity” OR (MH “ROC Curve”)
13. “sensitivity”
14. “specificity”
15. S7 OR S8 OR S9 OR S10 OR S11 OR S12 OR S13 OR S 14
16. S6 AND S15
17. “Symptom diagram” or “hand diagram” or “Flick sign” or “Provocative Test*” or “Phalen’s test” or “phalen test” or “wrist flexion test” or “wrist extension test” or “reverse Phalen test” or “carpal compression test” or “Durkan’s test” or “Tinel’s sign” or “Tourniquet test” or “Gilliat test” or “Sensory test*” or “Motor Test*” or “Touch” or “vibration threshold” or “Current perception threshold” or “Two-point discrimination” “Semmes-Weinstein Monofilament Test” or “Thenar weakness” or “Thumb Abduction Weakness” or “thenar atrophy” or “Abductor Pollicis Brevis Manual Muscle Testing” or “CTS-Relief Maneuver” or “CTS-RM” or “Pin Prick Sensory Deficit” or “ULNT Criterion C” or “upper limb neurodynamic test” “Tethered median nerve stress test” or “Luthy’s sign” or “luthy sign” or “scratch collapse test” or “Pinwheel” or “CTS-6 evaluation tool” or “The Alderson-McGall hand function questionnaire” or “Hand elevation test” or “Katz and Stirrat hand diagram” or “katz hand diagram” or “Purdue Pegboard Test” or “Levine’s Self-Assessment Questionnaire” or “Dellon-modified Moberg pick-up test” or “Self-administered diagram” or “web-based questionnaire” or “Kamath and Stothard questionnaire” or “Lo Carpal Tunnel Questionnaire” or “scratch-collapse test” or “hyperextension test” or “Flinn Performance Screening Tool” or “FPST”
18. S16 AND S17

## Appendix B

**Table 7:** Conflicts of interest

Author/year	Conflict of interest
Borg & Lindblom [20] /1988	ND
Buch-Jaeger & Foucher [21] / 1994	NR
Dale et al. [8] /2011	NR
Franzblau et al. [22] /1993	NR
Katz et al. [23] /1990	Grant Support: By NIH Grants AR36308 and AR07530 and the Kellogg Program for Training in Research in Clinical Effectiveness
Kucukakkas & Yurdakul [7]/ 2019	No conflict of interest
Kuhlman et al. [24] /1997	NR
MacDermid et al. [25] /1994	NR
MacDermid et al. [26] /1997	NR
Makanji et al. [11] /2013	ND
Naranjo et al. [9] /2007	NR
Pagel et al. [27] /2002	NR
Raudino [28] /2000	NR
Sartorio [10] /2017	ND
Szabo et al. [29] /1999	ND
Yildirim & Gunduz [6] /2015	NR

List of abbreviations: *ND* none declared, *NR* not reported

## Appendix C

**Table 8:** Reference standards

Authors and year	Reference Standard Test	Reference Standard Test Methodology	Positive Results Criteria
**Borg & Lindblom 1988** [20]	Examined the efficacy of a combined battery of tests	Not described.	Not described.
**Buch-Jaeger & Foucher 1994** [21]	NCS	Four criteria were examined.	Nerve conduction studies were taken as positive when the distal motor latency in the abductor brevis muscle was greater than 4 ms and the speed of sensory nerve conduction through the carpal tunnel was less than 50 m/s.
**Dale et al. 2011** [8]	Modified Katz Hand Diagram	A team of three health professionals (two physicians and an occupational therapist) independently rated each Katz hand diagram as “Unlikely,” “Possible,” “Probable,” or “Classic” for CTS.	CTS symptoms of the hand defined as a “classic” or “probable” rating on the modified Katz hand diagram.
	NCS	Examiners performed median and ulnar sensory and motor nerve conduction studies at the wrist bilaterally using the NC-Stat automated nerve conduction testing device (NEUROMetrix, Inc., Waltham, MA). They calculated median-ulnar sensory latency difference (MUDS).	Abnormal median nerve conduction, defined as sensory latency > 3.5 ms (14 cm) or motor latency > 4.5 ms or MUDS of > 0.5 ms (14 cm).
	The Consensus Criteria CTS Case Definition	CTS Symptoms Plus Abnormal Median Nerve Conduction.	A CTS case definition drawn from the consensus criteria of Rempel et al. [1998] requiring both symptoms (a “classic” or “probable” rating on a modified Katz hand diagram) and abnormal median nerve conduction (as defined above).
**Franzblau et al. 1993** [22]	1) NCS	Bilateral limited electrophysiologic testing of the median and ulnar nerves at the wrists. Measured parameters included sensory amplitude, peak latency and takeoff latency in each nerve tested.	A difference of at least 0.5 milliseconds between median and ulnar sensory peak latencies in the same wrist.
	2) NCS + various surveillance symptom definitions for CTS	The self-administered questionnaire focused on demographic information, prior medical conditions, occupational history, current health status, and symptoms which may be related to upper extremity cumulative trauma disorders.	Eight CTS cases were defined.
	3) Physical examination findings combined with various surveillance symptom definitions for CTS	The physical examination included inspection, palpation, active and passive range of motion of joints, elicitation of reflexes (biceps, triceps, and brachioradialis), Tinel’s test, Phalen’s test, Finkelstein’s test, and 2-point discrimination.	NR
**Katz et al. 1990** [23]	NCS	The protocol included bilateral median and ulnar sensory and motor testing and electromyographic recording from the abductor pollicus brevis on the most symptomatic hand. Testing was done with standard techniques on a Disa 1500 (Copenhagen, Denmark) or Teca 42 (Pleasantville, New York) apparatus.	If patients had median motor latency greater than 4.0 ms, sensory latency greater than 3.7 ms, or sensory velocity less than 50 m/s. performed by neurologist
**Kucukakkas & Yurdakul 2019** [7]	NCS	All the electrophysiological examinations were performed according to the American Association of Electrodiagnostic Medicine (AAEM) guidelines for CTS by one examiner using a Neuropack S1 MEB 9400 (Nihon Kohden Corporation, Tokyo, japan).	AAEM guidelines
**Kuhlman et al. 1997** [24]	NCS	Six different NCS methods were performed on all 228 hands.	In addition to the subjective symptoms of CTS, one of the three objective electrodiagnostic criteria must have been met for a patient to be diagnosed with CTS.
**MacDermid et al. 1994** [25]	Clinical profile of CTS	The electrodiagnostic testing was performed in the hospital laboratory using the laboratory standards for abnormality of median nerve conduction velocity and/or distal sensory latency.	A clinical profile of CTS determined by hand surgeons based on history and gross motor and sensory inspection, combined with independently obtained electrodiagnostic evidence of CTS.
**MacDermid et al. 1997** [26]	NCS and positive clinical examination from experienced hand surgeons	Physical examination included detailed history of symptoms and aggravating factors, two-point discrimination and light touch sensory evaluation and strength testing of abductor pollicis brevis by manual muscle testing. Nerve conduction tests and electromyographic testing performed by blinded staff neurologists.	Evaluation of normality was considered in the context of the entire neurophysiologic examination, which induced testing of ulnar, radial and proximal nerves as sources of pathology and examination of distal latencies, amplitudes and conduction times for motor and sensory nerves.
**Makanji et al. 2013** [11]	NCS	All of the patients had electrophysiological testing (nerve conduction velocity and electromyography) in the same office. Median nerve conduction studies were performed across the wrist.	standards based on the American Association of Neuromuscular and Electrodiagnostic Medicine (AANEM).
**Naranjo et al. 2007** [9]	NCS	Tests were performed with the guidance of two neurologists following the American Academy of Neurology protocol. These include performing a median sensory or motor nerve conduction studies.	An initial latency over 3.4 ms was considered abnormal.
**Pagel et al. 2002** [27]	NCS	All electrodiagnostic testing was performed with a Nicolet Viking IV D (Nicolet, Madison, WI). The median and ulnar nerves were stimulated in the palm, and the response was recorded 8 cm proximally at the wrist.	If a patient had a median ulnar latency difference of 0.3 msec or an absent median response and a normal ulnar response.
**Raudino 2000** [28]	NCS	motor latencies of median and ulnar nerve were recorded using surface electrodes placed over the abductor pollicis brevis and abductor digiti minimi respectively, and stimulating supramaximal at the wrist at a distance of 6 cm.	According to their normal values (mean + 2 SD), latencies greater than 3 ms were considered abnormal.
**Sartorio 2017** [10]	NCS	Subjects with suspected CTS was subdivided into 4 groups based on EMG (severe/extreme-GrA, moderate-GrB, mild/minimal-GrC, negative-GrD)	The presence of CTS was defined as positive EMG (GrAGrC), while subjects with negative EMG included in the GrD were considered healthy.
**Szabo et al. 1999** [29]	NCS	Bilateral median and ulnar motor and sensory nerve conduction testing were the electrodiagnostic parameters considered in this study.	Abnormal if the latency was ≥4.5 ms or ≥ 3.5 ms across the wrist, respectively. If either one or both were abnormal, the patient was considered to have a positive electrodiagnostic test.
Yildirim & Gunduz 2015 [6]	NCS	The instrument used was a Medelec Sapphire 4 ME. Bilateral median motor and sensory nerve conduction potentials were recorded using standard techniques according to the practice parameters for the electrodiagnosis of CTS outlined by the American Academy of Neurology, the American Association of Neuromuscular and Electrodiagnostic Medicine, and the American Academy of Physical Medicine and Rehabilitation.	Abnormal electrophysiological findings suggesting CTS were categorized into three grades according to Stevens’ classification: mild, moderate, and severe.

## Abbreviations

CTS: Carpal tunnel syndrome
SR: Systematic review
Sn: Sensitivity
Sp: Specificity
+LR: Positive likelihood ratio
-LR: Negative likelihood ratio
PPV: Positive predictive value
NPV: Negative predictive value
NCV: Nerve conduction velocity
AAOS: American Association of Orthopedic Surgeons
